# Antibiotic overuse as a modifiable early-life risk factor for non-communicable diseases in sub-Saharan Africa

**DOI:** 10.1080/16549716.2026.2646042

**Published:** 2026-03-18

**Authors:** Michelle Leal, Johanni Beukes, Stephanie Alcock, Urlridge Thompson, Shane A. Norris

**Affiliations:** a SAMRC/Wits Developmental Pathways for Health Research Unit, Department of Paediatrics, Faculty of Health Sciences, School of Clinical Medicine, University of the Witwatersrand, Johannesburg, South Africa; b School of Human Development and Health, Faculty of Medicine, University of Southampton, Southampton, UK

**Keywords:** Gut microbiota, antimicrobial stewardship, developmental origins, life-course epidemiology, health policy

## Abstract

Early-life antibiotic overuse is a public health concern. In low- and middle-income countries, consumption has surged by 76% since 2000. This trend is particularly acute in sub-Saharan Africa (SSA), where antimicrobial resistance contributes to 255,000 deaths annually and infant antibiotic exposure is widespread in the first two years of life. While a substantial body of research associates antibiotic-induced microbiome disruption with metabolic and immune dysregulation, with large cohorts reporting ~20% higher odds of childhood obesity and asthma, these observational findings do not establish causality and derive largely from high-income settings. This potential pathway remains a policy blind spot within most non-communicable disease (NCD) prevention frameworks. By synthesising biological, epidemiological and implementation evidence, this paper considers early-life antibiotic exposure as a potentially modifiable determinant of lifelong health and outlines a pragmatic research and policy agenda to integrate antibiotic-aware prevention into NCD prevention efforts and routine child health platforms in resource-limited SSA settings.

## PAPER CONTEXT

**Main findings:** Pervasive early-life antibiotic use, driven by rising consumption in LMICs and limited stewardship in sub-Saharan Africa, represents a significant and underappreciated driver of non-communicable disease risk.

**Added knowledge:** This paper bridges the policy gap between strategies to contain antimicrobial resistance and strategies to prevent chronic non-infectious diseases, by synthesising mechanistic, epidemiological and implementation evidence to reframe early-life antibiotic exposure as a primary, modifiable determinant of lifelong health.

**Global health impact for policy and action:** Health systems can address the dual burden of antimicrobial resistance and chronic non-infectious diseases by integrating antibiotic-aware risk profiling into routine child health, using existing platforms to track exposure and deliver targeted preventive interventions in high-burden settings.

## Background

Non-communicable diseases (NCDs), including cardiovascular disease, diabetes, chronic respiratory disease and cancer, account for approximately 74% of deaths worldwide annually [1]. In sub-Saharan Africa, the burden of NCDs has risen substantially; disability-adjusted life-years attributable to NCDs increased from about one-fifth of the total burden in 1990 to nearly one-third by 2017 [2] and NCDs are projected to account for roughly half of all deaths in many countries by 2030 [2,3]. In South Africa, NCDs cause more than half of all deaths [4]. Compounding this burden, antimicrobial resistance (AMR) is a parallel crisis: in 2019, it was associated with an estimated 4.95 million deaths worldwide, including 255,000 in SSA and around 40,000 in South Africa [5,6].

Antibiotic consumption in low‐ and middle‐income countries rose by 76% between 2000 and 2018 [7]. In SSA hospitals, antibiotics are prescribed for 28–84% of inpatients and 11–95% of paediatric admissions [8], while community‐level access is often poorly regulated. In several SSA settings, including Cameroon and South African hospitals, nearly half of antibiotics are dispensed without a prescription and broad‐spectrum agents such as ceftriaxone, amoxicillin – clavulanate and metronidazole account for about one‐third to one‐half of inpatient courses [9,10]. Although pharmacist‐led stewardship initiatives at South African tertiary centres have reduced inappropriate prescribing [10], stewardship efforts outside these centres remain uneven, and national AMR surveillance is incomplete [11,12]. Pervasive antibiotic use and limited stewardship are particularly concerning during infancy, when gut microbiota and immune systems are developing [13].

The developmental origins of health and disease (DOHaD) framework posits that exposures from conception through infancy set lifelong risk trajectories [14]. Antibiotics given in the first year can disrupt gut‐microbiota assembly at critical windows for immune and metabolic maturation [13,15]. In high‐income cohorts, such disruptions are associated with roughly 20% higher odds of childhood overweight by age seven and increased risk of asthma and other immune-mediated conditions [16,17]. These studies are observational and remain vulnerable to residual confounding, and there are almost no equivalent data from SSA. Despite high infant antibiotic exposure across SSA, no longitudinal cohort has yet linked early microbiome perturbations or infant antibiotic use to adult NCD outcomes [18]. This lack of cohort data from SSA represents a central evidence gap. The sections that follow summarise mechanistic and epidemiological evidence, highlight key gaps in the SSA evidence base and review stewardship examples to consider how antibiotic-aware prevention might be incorporated into existing maternal and child health platforms.

## Mechanistic pathways

Antibiotic administration during infancy can disrupt gut‐microbiota assembly at a critical juncture for immune and metabolic development [13,15]. Early colonisers, such as *Bifidobacterium* and *Faecalibacterium prausnitzii*, nurture barrier integrity and regulate short‐chain fatty acid production, which support epithelial health and modulate host metabolism [19,20]. In a Finnish birth cohort, β‐lactam antibiotics reduced *Bifidobacterium* abundance by up to 70% [21]. Those newborns also had ~45% higher cord‐blood interleukin‐6 - a marker of systemic inflammation. Animal models mirror these disruptions. Neonatal mice treated with low‐dose penicillin exhibited a 70% decline in *Faecalibacterium prausnitzii*, a key butyrate producer [22]. Within weeks, these mice showed a 15% increase in adiposity and early markers of insulin resistance. Antibiotic-induced shifts affect both Gram-positive and Gram-negative taxa; shifts in lipopolysaccharide-producing Gram-negative bacteria can promote low-grade systemic inflammation [13]. In human cohorts, antibiotic-associated shifts in microbiota composition and diversity can persist for several months, particularly after repeated broad-spectrum courses [21], suggesting that both exposure and duration of use are likely to be relevant to downstream effects.

Beyond short‐chain fatty acids, antibiotic‐induced dysbiosis rewires bile acid metabolism. Commensal bacteria convert primary bile acids into secondary forms that engage the farnesoid X receptor (FXR) and Takeda G‐protein coupled receptor 5 (TGR5), which regulate hepatic glucose output and lipid storage [15]. In a randomised crossover trial, seven days of oral vancomycin reduced secondary bile acids by 60% and increased the Homeostatic Model Assessment of Insulin Resistance (HOMA‐IR) index by 35%, indicating a substantial, short-term rise in insulin resistance [23,24].

Antibiotic‐driven microbiome changes also influence immune maturation. Loss of butyrate-producing taxa after antibiotic exposure shifts the immune balance towards pro-inflammatory T helper 17 (Th17) cells and away from regulatory T cells (Treg) [20], and in the Generation R cohort, infant antibiotic exposure was associated with lower stool butyrate and higher methylation of the forkhead box P3 (FOXP3) locus, a transcription factor critical for Treg development [21]. In mice, treating newborns with vancomycin reduced gut microbial diversity, lowered FOXP3^+^ Treg counts, and produced higher Th17 cytokines and worse allergic airway inflammation [25]. Together, these epigenetic and immune shifts provide a plausible pathway linking early-life antibiotics to later asthma, eczema and other immune-mediated disorders [17].

In SSA infants, baseline gut‐microbiota diversity is lower than in high‐income settings. Malawian 0–24-month‐olds exhibit mean Shannon indices around 1.5 versus ≈2.1 in US peers [26]. In Malawian twin pairs discordant for kwashiorkor, a form of severe acute malnutrition, undernourished siblings had markedly lower diversity than their healthy co-twins – and both groups fell below US diversity [26,27]. When antibiotics enter this context, loss of keystone taxa could amplify effects on metabolic programming. Mechanistic pathways have not yet been characterised in SSA infants. Evidence from animal models and high-income birth cohorts indicates that antibiotic-induced disruptions during infancy set the stage for metabolic and immunological dysregulation, but the contribution of these pathways to long-term NCD risk in SSA populations remains speculative and will require testing in region-specific longitudinal cohorts.

Table 1 summarises the main biological pathways discussed in this section, linking early-life antibiotic exposure, disruption of the gut microbiota and intermediate metabolic and immune changes with later NCD risk.

**Table 1.** Mechanistic pathways linking early-life antibiotic exposure to NCD risk.

System affected	Antibiotic-induced disruption	Downstream biological consequence	Potential NCD outcome
Metabolic Regulation *(via Short-Chain Fatty Acids)*	Reduction of early-life keystone gut bacteria (e.g. *Bifidobacterium*, *Faecalibacterium prausnitzii*) with broad-spectrum antibiotic use.	Lower butyrate production with altered lipid and mitochondrial metabolism; low-grade systemic inflammation (e.g. higher IL-6).	Childhood overweight and obesity; insulin resistance and type 2 diabetes.
Metabolic Regulation *(via Bile Acids)*	Reduced conversion of primary to secondary bile acids by the gut microbiota.	Reduced FXR and TGR5 signalling with dysregulated hepatic glucose output and lipid storage; higher HOMA-IR.	Insulin resistance, impaired glucose tolerance and metabolic syndrome.
Immune System Maturation	Loss of butyrate-producing bacteria and a shift in the immune cell balance.	Reduced regulatory T cells (Tregs), partly via increased FOXP3 methylation; expansion of pro-inflammatory Th17 cells and heightened inflammatory tone.	Childhood asthma, eczema and other immune-mediated inflammatory disorders.

*Note: Evidence is drawn from in vitro, animal, and human cohort studies* [13,15,19–27].

## Epidemiological links

High-income cohorts demonstrate long-term associations of infant antibiotics and later outcomes. In the United States (*n* = 11 000), any course before six months conferred 1.21-fold higher odds of overweight at age seven (95% CI 1.13–1.30) [16]. In Finland (*n* ≈ 12 000), antibiotic exposure in the first year was linked to BMI z-score increases at age two (+0.20 SD for boys; +0.13 SD for girls) [28]. A meta-analysis of 14 birth cohorts found early antibiotics raise asthma risk by 1.18-fold (95% CI 1.09–1.27) and eczema risk by 1.15-fold (95% CI 1.06–1.24) [17]. These estimates derive from observational birth cohorts in high-income settings and remain susceptible to residual confounding, with no published SSA birth cohorts linking infant antibiotic use to later NCD outcomes [18].

Antibiotic-mediated microbiome perturbations may follow conserved pathways across populations; however, whether effect sizes in SSA approximate those seen in high-income cohorts is unknown and requires empirical evaluation [13,17]. The overlapping burdens of undernutrition, where 30% of children under five in SSA are stunted [29], and rapid rises in antibiotic consumption [7] may amplify metabolic and immune dysregulation beyond high-income projections. Incorporating antibiotic history into routine child-health records may sharpen risk stratification and identify infants at higher risk of obesity, diabetes and cardiovascular disease.

## Framing the debate: traditional non-communicable disease prevention vs. integrating antibiotic exposure

Current chronic-disease strategies largely focus on established adult behavioural risk factors, such as diet, physical activity and tobacco use [30], and South Africa’s National Department of Health NCD Strategic Plan 2022–2027 makes no mention of early-life microbial exposures [31]. Antimicrobial resistance strategies have, out of necessity, focused almost exclusively on resistance containment, without addressing the downstream metabolic or NCD consequences of antibiotic use [32].

While both perspectives are critical, their separation creates a policy gap. Taken together, the mechanistic and epidemiological evidence suggests that early-life antibiotic exposure may be an important, modifiable early-life determinant of NCD risk, although direct causal pathways and effect sizes in sub-Saharan Africa remain to be established.

This reframing is not merely conceptual; it translates into pragmatic, actionable shifts in routine child health. Integration can be achieved by leveraging existing maternal and child health platforms. Adding antibiotic-tracking fields to routine child health records, such as South Africa’s Road to Health booklet, would be a cost-effective first step. Based on this data, infants with significant exposure (e.g. ≥2 courses before 12 months) could be flagged for targeted interventions, including microbiome-supportive counselling and focused caregiver education on judicious antibiotic use. Community health workers, already a cornerstone of primary care in the region, could be trained to reinforce these messages during home visits. In practice, introducing new data elements into child health records and community workflows requires governance, training and data-management capacity, and experience from other programmes (e.g. immunisation and growth monitoring) shows that such changes are feasible but not trivial in low-resource settings.

As summarised in Table 2, this integrated approach reframes core pillars of NCD prevention. Routine child health encounters become risk-profiling opportunities, community health workers become agents of both stewardship and NCD prevention, and antibiotic-exposure records can be used within health information systems to identify high-risk infants.

**Table 2.** Integrating antimicrobial stewardship into non-communicable disease prevention: reframing core pillars to include antibiotic tracking.

Domain	Traditional focus	With antibiotic tracking
Risk profiling	Age, blood pressure, body‐mass index	Early‐life antibiotic history, microbiome markers, social risk
Interventions	Diet, exercise, tobacco control	Microbiome‐supportive counselling, caregiver education, CHW stewardship
Surveillance	Periodic screening camps, clinic registers	Antibiotic-exposure flags in child-health records; linked dashboards
Evidence generation	Lifestyle trials, pharmaco-epidemiology	Stewardship implementation trials; birth-cohort microbiome – NCD linkage

## Illustrative case studies

Case studies illustrate how antibiotic-tracking levers can operate in practice, although most examples to date are pilot or programme-level interventions rather than fully scaled national strategies.

Sweden’s national Strategic Programme Against Antibiotic Resistance (Strama) combined updated paediatric guidelines, public-awareness campaigns and prescriber feedback. Outpatient paediatric prescriptions fell from 266 to 194 per 1000 inhabitants between 2006 and 2014 (mean annual change − 8.5; 95% CI −11.9 to −5.2) [33].

In England, a pragmatic national randomised trial sent ‘social-norm’ feedback letters to 1500 general practices identified as high antibiotic prescribers. Over six months, these practices cut antibiotic items per 1000 patients by 3.3% compared with controls (difference-in-differences; 95% CI −5.8 to −0.8; *p* = 0.005) [34].

In Tanzania, the ALgorithm for the MANagement of Childhood Illness (ALMANACH) smartphone tool was evaluated in 1465 children aged 2–59 months. On day 0, antibiotic prescriptions dropped from 84.3% under standard practice to 15.4% with ALMANACH – a relative reduction of 82% [35].

At a tertiary hospital in Malawi, a pharmacist-led stewardship programme – including prescription audits, feedback sessions and locally tailored guidelines – reduced third-generation cephalosporin use by 27% over 12 months (200 → 146 defined-daily-doses per 1000 patient-days; *p* < 0.01) and saved USD 15,000 in antibiotic costs [36].

Across 47 South African hospitals, antimicrobial-stewardship committees raised guideline-concordant prescribing from 62% to 80% and cut inappropriate broad-spectrum use by 28% over two years [10].

These pilots cover national policy, digital decision support and facility‐level stewardship bundles. They demonstrate that specific levers – such as feedback, clinical decision support and multidisciplinary stewardship teams – can reduce antibiotic prescribing in diverse settings. However, evidence on long-term sustainability and national scale-up, particularly in resource-limited SSA health systems, remains limited. Adapting these approaches to routine child-health platforms will require attention to workforce capacity, financing and health-information systems if they are to support antibiotic-aware NCD prevention.

## Implementation barriers and systemic constraints

Translating successful pilots into routine practice requires overcoming significant systemic constraints.

First, fundamental gaps in surveillance hinder effective risk profiling. Current platforms, such as the WHO’s GLASS, report antibiotic volumes at the national level but lack a child-level registry to track individual courses [37]. Without individual-level exposure data, infants at higher risk cannot be identified, blunting the potential of a targeted prevention strategy.

Second, the evidence base itself has limitations. Mechanistic data derive largely from high-income contexts, and it remains unclear how these pathways translate to SSA, where diet, co-infections, and environmental factors differ markedly [38]. Furthermore, few cohorts in the region have the established infrastructure or personnel for the high-throughput microbiome and multi-omic analyses required to generate local evidence.

Finally, challenges at the primary care and community level complicate both surveillance and intervention. The pervasive practice of antibiotic self-medication, with nearly half of antibiotics in some settings obtained without a prescription [9], means a substantial proportion of use occurs outside the formal health system. This is compounded by stewardship efforts that remain largely hospital centric. While stewardship committees are functional in many tertiary centres [11], primary-care clinics, the front line of paediatric care, often lack formal AMS frameworks and support [12]. Together, these factors create an environment where judicious antibiotic use is difficult to promote or monitor.

## Discussion

Early-life antibiotic exposure is a potentially modifiable determinant of lifelong health, supported by three strands of evidence. The biological pathway is plausible, as animal and in vitro models consistently show that antibiotics disrupt microbial processes essential for normal metabolic and immune development [15,22]. Epidemiological data from large birth cohorts in high-income countries provide further support, linking infant antibiotic use to a higher risk of childhood obesity and allergic conditions [16,17]. Finally, the pragmatic feasibility of intervention is demonstrable; case studies from diverse settings show that well-designed stewardship levers can reduce paediatric prescribing by approximately 25% to over 80% [33,35].

However, the limitations of the evidence base should be acknowledged, particularly within the sub-Saharan African context. The synthesis of developmental-origins biology, large birth cohorts and stewardship pilots highlights a plausible pathway from early-life antibiotic exposure to later NCD risk, but critical gaps remain. The epidemiological links are, to date, correlational and primarily from high-income settings, leaving them susceptible to residual confounding [18]. The absence of a longitudinal SSA cohort directly linking infant antibiotic exposure to adult NCD outcomes remains the most significant evidence gap. This is compounded by the systemic barriers previously discussed – including fragmented surveillance and unregulated community-level antibiotic access – which complicate both measurement and intervention [9,12,37].

Addressing these complexities requires that policy and research agendas advance in tandem. For policy, sustained antibiotic-aware prevention requires action. First, surveillance must be strengthened by embedding antibiotic-exposure fields within routine child-health records. Second, combining digital decision-support, such as the ALMANACH algorithm, with community-health-worker stewardship training can reinforce judicious use [35]. Finally, national guidelines should adopt WHO’s AWaRe framework to embed stewardship principles into all levels of prescribing practice [39].

The main research priority is to address these gaps. Longitudinal sub-Saharan African birth cohorts that document early-life antibiotic exposure and follow metabolic and immune outcomes are needed [18], with embedded microbiome and other multi-omic profiling to test mechanistic pathways in local contexts [38]. Pragmatic implementation trials should in parallel assess the effectiveness, costs and equity impacts of stewardship strategies in routine care. Although this commentary focuses on infancy, antibiotic stewardship later in life, including in older adults, is also likely to be relevant for NCD prevention but lies beyond its scope. Such work will require sustained national and international investment through agencies that support research on antimicrobial resistance and chronic disease in low- and middle-income countries, including national research councils and global health funders.

In conclusion, tackling the dual burden of infectious and non-communicable diseases in SSA demands an integrated prevention framework. By aligning pragmatic policy shifts with a rigorous, context-specific research agenda, early-life antibiotic stewardship can be elevated from an underappreciated risk factor to a cornerstone of NCD prevention.
